# Acute oral toxicity, apoptosis, and immune response in nurse bees (*Apis mellifera*) induced by flupyradifurone

**DOI:** 10.3389/fphys.2023.1150340

**Published:** 2023-03-28

**Authors:** Jing Gao, Yi Guo, Jin Chen, Qing-Yun Diao, Qiang Wang, Ping-Li Dai, Li Zhang, Wen-Min Li, Yan-Yan Wu

**Affiliations:** ^1^ State Key Laboratory of Resource Insects, Institute of Apicultural Research, Chinese Academy of Agricultural Sciences, Beijing, China; ^2^ College of Life Sciences and Agriculture and Forestry, Qiqihar University, Qiqihar, China

**Keywords:** flupyradifurone, nurse bees, acute toxicity, mushroom bodies, apoptosis, immune

## Abstract

The potential toxicity of flupyradifurone (FPF) to honey bees has been a subject of controversy in recent years. Understanding the effect of pesticides on nurse bees is important because the fitness of nurse bees is critical for in-hive activities, such as larval survival and performing hive maintenance. In order to evaluate the acute oral toxicity of flupyradifurone on nurse bees, flupyradifurone at five different concentrations was selected to feed both larvae and nurse bees. Our results showed that nurse bees were more sensitive to flupyradifurone than larvae (LD_50_ of the acute oral toxicity of flupyradifurone was 17.72 μg a.i./larva and 3.368 μg a.i./nurse bee). In addition, the apoptotic rates of neurons in mushroom bodies of nurse bees were significantly induced by flupyradifurone at sublethal concentrations (8 mg/L, 20 mg/L, and 50 mg/L) and the median lethal concentration LC_50_ (125 mg/L). The expression of immune-related genes (*Hsp90*, *Toll-8/Tollo*, and *defensin*) was significantly changed in exposed nurse bees at the field-realistic concentration of flupyradifurone. However, three detoxifying enzyme genes (*CYP9Q1*, -2, and -3) were not affected by pesticide exposure. Our data suggest that although flupyradifurone had a relatively lower acute oral toxicity than many other common pesticides, exposures to the field-realistic and other sublethal concentrations of flupyradifurone still have cytotoxicity and immune-responsive effects on nurse bees. Therefore, flupyradifurone should be considered for its application in crops.

## Introduction

Flupyradifurone (FPF) is a systemic butenolide insecticide that was first registered commercially in 2014. It has a lower binding affinity to insect nAChRs than neonicotinoids, and it is effective against numerous neonicotinoid-resistant insects (Glaberman and White, 2014; Nauen et al., 2014; Chen et al., 2017). In the wild, honey bee foragers may carry pesticide-contaminated nectar or pollen back to the colony. These pesticide-laden foods can harm not only the foragers themselves, but also the larvae through the nurse feeding behavior (Medrzycki et al., 2003; Forfert and Moritz, 2017; Abou-Shaara et al., 2021; Chen et al., 2021; Wueppenhorst et al., 2022). Young bees (hive bees) between 3 and 13 days old were termed as nurse bees, whose tasks were mainly processing food and provisioning bee larvae. They feed pure royal jelly to the queen larva and a mixture of pollen, honey, and jelly to the worker and drone larvae (Lindauer and Watkin, 1953; Metz et al., 2021). Pesticides would affect nurse bees and cause larval deaths due to insufficient care (Yang et al., 2012; Guo et al., 2021). Moreover, previous studies indicated that the toxicity of several pesticides (imidacloprid, thiamethoxam, and formetanate) to adult bees was higher than that to larvae (Yang et al., 2012; Laurino et al., 2013; Tavares et al., 2015; Dai et al., 2017; Staroň et al., 2017). Nurse bees in the early stages of adulthood seemed to be more sensitive to pesticides. Comparison of the acute toxicity of FPF to honey bee larvae and nurse bees was imperative.

In addition to acute toxicity, the cytotoxicity of pesticides to honey bees could also be detected by an apoptotic assay. The TUNEL [terminal deoxynucleotidyl transferase (TdT)-mediated dUTP Nick-End Labeling] method was used to detect apoptosis, which enabled visualization and quantification of apoptotic cells. In our previous study, significant neuronal apoptosis was observed in the honey bees’ brains after exposure to the sublethal doses of imidacloprid for 3–12 days (Wu et al., 2015). Chakrabarti et al. (2020) reported a significant increase in the activity of caspase-3 protein (an indicator of the onset of apoptosis) in honey bees after FPF treatment (a 10-day contact exposure of formulated FPF at the field rate) but not for 6-h treatment. However, there were some pesticide adjuvants in the formulated FPF which affected the exact effect of FPF. Little has been reported on the effect of pure FPF in the central nervous system of nurse bees.

Certain sublethal effects were not apparent or external, but they could cause molecular-level damage. Potential toxic effects of pesticides included changes in gene and protein expressions, as had been reported in previous studies (Wu et al., 2015; McKinstry et al., 2017; Tavares et al., 2019; Hou et al., 2020; Naggar and Paxton, 2020; Guo et al., 2021). In our previous research, after 6-day exposure on colonies and 12-h exposure of acute toxicity on newly emerged honey bees, FPF at field-realistic concentrations was reported to induce immune challenge, detoxification response, and olfactory learning deficits through different expression of certain genes in adult bees, especially in young bees (Wu et al., 2015; Guo et al., 2021).

Given the importance of nurse bees, this study was first designed to investigate the acute oral toxicity of FPF to nurse bees and rearing larvae *in vitro*, with the goal of determining whether FPF was more toxic to nurse bees than to larvae. The apoptosis of neurons in the mushroom bodies of nurse bee brains was measured in order to investigate the effects of FPF on the target site of nurse bees. After that, the effects of FPF on the expression of genes related to detoxification and immune response were detected.

## Methods and materials

All experiments were conducted in the Institute of Apicultural Research, Chinese Academy of Agricultural Sciences (Beijing, China). Also, 10 healthy and strong colonies (*A. mellifera*) used in this study were maintained in an apiary of the institute (it was 8 weeks from the time bees were treated with chemical substances).

### Acute oral toxicity test on larvae

Larval diets and chemical preparation: There are three different larval diets (diets A, B, and C) (OECD, 2013; Schmehl et al., 2016). Each diet components were mixed in the following order until they were dissolved completely: 4.43 g of filtered ddH_2_O, 0.53 g of D-fructose and D-glucose, 0.09 g of yeast extract, and 4.43 g of royal jelly for diet A. Diet B comprised 4.40 g of filtered ddH_2_O, D-fructose and D-glucose (0.64 g), yeast extract (0.13 g), and royal jelly (4.30 g); and diet C comprised 15.00 g of filtered ddH_2_O, D-fructose and D-glucose (4.50 g), yeast extract (1.00 g), and royal jelly (25.00 g).

Flupyradifurone (purity 99.5%, Chem Service Inc., West Chester, PA, United States) was dissolved in sterilized ddH_2_O to prepare stock solutions (stored at 4°C), and the rate of the tested solution in diet C was 2% of the final volume. The following concentrations of FPF used for the larval assay were 125, 250, 500, 1,000, and 2000 mg L^−1^ (FPF is a water-miscible compound).

Larval rearing and FPF exposure: The protocol for the *in vitro* rearing of honey bee larvae was according to OECD (2013) and Schmehl et al. (2016). Five queens were kept on an empty comb in five hives for 24 h, and newly laid eggs were tagged. Then, 72 h after the queens were released, the successfully hatched eggs were grafted in the laboratory. For the sterile tissue culture plates (STCPs), 20 μL of diet A was added to the bottom of each cell cup. Then, 12 young larvae of the same day (hatched within 24 h) on each comb were randomly transferred to the STCP as one replicate, and they were quickly transferred to incubators at 35°C ± 0.5°C and 94% relative humidity. There were five replicates (colonies) per treatment, with 12 larvae per replicate (60 larvae per treatment). After 48 h (days 1 and 2) of feeding on diet A, each larva was fed 20 μL diet B on day 3. On day 4, each larva was fed 30 μL diet C containing FPF at five different concentrations (final concentration of FPF in diet C was 125, 250, 500, 1,000, and 2000 mg L^−1^; control was fed FPF-free diet C), and each larva on days 5 and 6 was fed 40 μL and 50 μL diet C. The number of dead larvae was examined and recorded at the same timepoints on days 5, 6, and 7 (the larvae at the termination of the test were before the pre-pupal stage). The aforementioned operations are carried out in a positive flow-sterilized hood. The cumulative mortality of larvae on day 7 was calculated. LC_50_ was obtained according to the method of Dai et al. (2017). The concentration–response curves at 72 h were used to determine the median lethal concentration by probit analysis (Finney, 1971). Then, the median lethal dose was calculated since each larva was fed with a 30 μL diet containing FPF on day 4.

### Acute oral toxicity test on nurse bees

Chemical preparation: A total of five FPF concentrations (31.25, 62.5, 125, 250, and 500 mg/L) were based on our previous experiments. The FPF stock solutions were gradually diluted with 50% sucrose solution into the aforementioned final concentrations and stored at 4°C.

Rearing of nurse bees and FPF exposure: The protocol was modified from the OECD (1998) for *A. mellifera*. To obtain the nurse bees of similar age, three broods along with sealed worker bees from three colonies, which were different from those in the larval assays, but the same in apiary, were placed in an incubator at 30°C and 65% humidity. The newly emerged bees within 12 h were randomly captured into cages on the next day. Each cage contained 20 bees as a group of replicates, and each treatment contained four replicates (80 bees for each test concentration). The bees were fed with 50% (w/v) sucrose solution (changed every day) *ad libitum* for 1 week. The bees were starved for 2 h before the initiation of the experiment.

The treatment groups were fed with 50% sucrose solution containing the aforementioned five FPF concentrations. The control groups were fed with sucrose solution without FPF. After feeding for 24 h, the number of dead bees was checked and recorded. Adjusted mortality and the median lethal concentration were obtained by referring to the larval experiment. Then, a food consumption assay for FPF exposure at LC_50_ was conducted to obtain LD_50_.

### Apoptosis detected by TUNEL

The procedure was modified from previous studies (Chen et al., 2014; Wu et al., 2015). In brief, brains of anesthetized honey bees were dissected into 4% paraformaldehyde for 4 h, dehydrated in 30% sucrose solution for 12–15 h, embedded in tissue freezing medium, and then cut into frozen tissue slices (7–10 μm thick). The slices were fixed on the Superfrost Excell slides, permeabilized with 0.2% Triton X-100 for 10 min, and then incubated with fetal bovine serum for 30 min. The frozen slides were incubated with TUNEL solution in the dark at 37°C for 4 h and then stained with DAPI for 6 min. They were washed twice in PBS (10 min × 2) between each two steps mentioned previously. Fluorescence was observed using a Leica fluorescence microscope. ImageJ 1.53f51 software was used to count the neurons in blue color and the apoptotic cells in green color on the slices (Gahm et al., 2021).

Apoptosis rate analysis: Three bees were randomly selected from each treatment, and five slices were cut from each brain, that is, each replicate (brain) contained five slices, which were added up to 400–500 neurons in the mushroom bodies, and more than 1,000 neurons were counted per treatment. The percentage of apoptotic cells was determined by comparing the number of DAPI-stained neurons to that co-labeled with TUNEL staining.

### qRT-PCR analysis

The effects of FPF on the relative expression of genes related to immune pathways and detoxification were examined. Based on the previous study, six genes for AMPs related to immune pathways (*defensin*, *hymenoptaecin*, *abaecin*, *Toll*, *Toll-8/Tollo*, and *Hsp90*) and three genes for detoxification (*CYP9Q1*, *CYP9Q2*, and *CYP9Q3*) were selected (Akhouayri et al., 2011; Wu et al., 2015; McKinstry et al., 2017; Yao et al., 2018; Balakrishnan et al., 2021; Guo et al., 2021). The methods for bee rearing, chemical exposure, and sample collection were the same as those used in the 4-mg/L treatment group of the apoptotic assay. After 24 h FPF exposure, the anesthetized survival bees were frozen with liquid nitrogen and stored at −80°C. The mixed tissues of five bees from each cage were set as one replicate for total RNA extraction, and a total of 15 bees were in each treatment. qRT-PCR procedures referred to our previous study (Wu et al., 2015; Guo et al., 2021). Briefly, total RNA was isolated using the TRIzol reagent; first-strand cDNA was synthesized following the manufacturer’s instruction. Quantitative RT-PCR and amplification were followed, as described by Guo et al. (2021). The relative expression was analyzed by the ΔΔCT method using *β-actin* as a reference gene (Livak and Schmittgen, 2001). Primers are shown in Table 1.

**TABLE 1:** Sequences of primers for the genes tested.

Gene name	Primer (5′ to 3′)	Reference
*defensin*	F: TGC​GCT​GCT​AAC​TGT​CTC​AG	Evans (2006)
	R: AAT​GGC​ACT​TAA​CCG​AAA​CG	
*hymenoptaecin*	F: CTC​TTC​TGT​GCC​GTT​GCA​TA	Evans (2006)
	R: GCG​TCT​CCT​GTC​ATT​CCA​TT	
*abaecin*	F: CAG​CAT​TCG​CAT​ACG​TAC​CA	Evans (2006)
	R: GAC​CAG​GAA​ACG​TTG​GAA​AC	
*Hsp90*	F: CAT​GGC​TAA​TGC​CGG​AGA​GG	McKinstry et al. (2017)
	R: CTG​CAC​CAG​CTT​GAA​GAG​C	
*Toll*	F: TCT​ATG​TTT​TGA​GCA​CCG​AGT	Ratiu et al. (2016)
	R: CAA​CGG​ATA​GTT​ATT​CGG​CCT	
*Toll-8*/*Tollo*	F: ACA​ATC​AGA​GGA​CCA​CGC​AG	This study
	R: AAG​CAA​CGA​AAC​GAA​GGT​GC	
*CYP9Q1*	F: TCG​AGA​AGT​TTT​TCC​ACC​G	Mao et al. (2011)
	R: CTCTTTCCTCCTCGATTG	
*CYP9Q2*	F: GAT​TAT​CGC​CTA​TTA​TTA​CTG	Mao et al. (2011)
	R: GTTCTCCTTCCCTCTGAT	
*CYP9Q3*	F: GTTCCGGGAAAATGAATC	Mao et al. (2011)
	R: GGTCAAAATGGTGGTGAC	
*β-actin*	F: TTG​TAT​GCC​AAC​ACT​GTC​CTT​T	Deng et al. (2020)
	R: TGG​CGC​GAT​GAT​CTT​AAT​TT	

### Statistical analysis

All data are presented as mean ± SEM. Mortality was analyzed by the probit method (Finney, 1971). The median lethal concentrations (LC_50_) with 95% confidence interval limits used the least-squares regression analyses of relative growth rates with the logarithm of the FPF concentration (Dai et al., 2017). The apoptotic rate of neurons was converted into square root arcsine. Therefore, significance for the apoptotic rate and the relative expression of genes were analyzed using ANOVA and Tukey HSD for multiple comparisons with IBM SPSS Statistics version 26.0, and the significance was defined as *p* < 0.05.

## Results

### Acute oral toxicity of FPF

We detected the LD_50_ value for bees at two early developmental stages (uncapped larva and nurse bees) and referred to the methods of OECD (OECD, 1998; OECD, 2013) to test the hypothesis that FPF was more toxic to nurse bees than to larvae. Five different FPF concentrations were separately fed to larvae (day 4 after the eggs hatched) in different treatment groups, and the adjusted mortality of larvae was calculated 72 h after exposure. The LC_50_ value for acute oral toxicity of a bee larva was 590.59 mg/L (y = 1.80x + 0.02 and *R* ^2^ = 0.9913), and its corresponding LD_50_ was 17.72 μg a.i./larva.

According to the adjusted mortality of nurse bees (7 days after emergency) and another five different FPF concentrations exposed for 24 h, the LC_50_ value for nurse bees was 125.92 mg/L (y = 3.94x−3.28 and *R* ^2^ = 0.9853). Since the average food consumption at this concentration was 26.75 μL/bee/day, the corresponding LD_50_ was 3.368 μg a.i./bee. Therefore, nurse bees were more sensitive and susceptible to FPF than larvae.

### Apoptotic rate of neurons in the mushroom bodies of nurse bees induced by FPF

**FIGURE 1:**
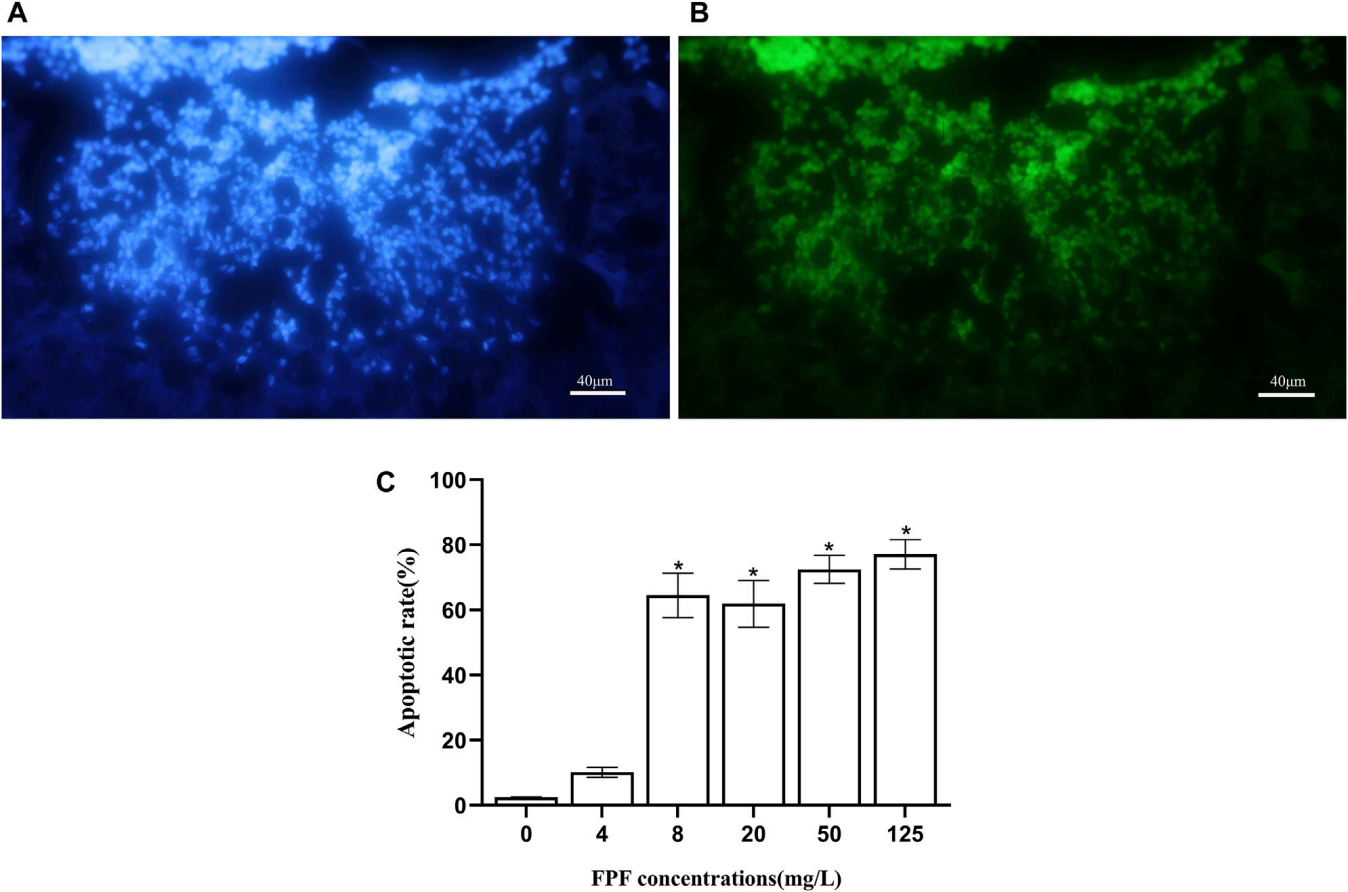
Flupyradifurone-induced apoptosis in neurons in the mushroom bodies of *Apis mellifera.* **(A)** Neurons stained by DAPI. **(B)** Apoptotic cells: TUNEL-positive cells. **(C)** Apoptotic rate of neurons in the mushroom bodies of *A. mellifera* induced by flupyradifurone.

Neurotoxin affects the central nervous system of insects. The mushroom bodies were the multi-functional center of the brain. The TUNEL method was used to detect the apoptosis of neurons in the mushroom bodies of nurse bees induced by FPF. The pesticides at 4 mg/L (field-realistic concentration), 8 mg/L, 20 mg/L, 50 mg/L, and 125 mg/L (LC_50_) were fed to nurse bees, which were reared as described in the acute toxicity assay. The neurons in blue color were stained using DAPI, as shown in Figure 1A, which showed the neurons in the mushroom bodies of nurse bees, and the neurons in green color, as shown in Figure 1B, were TUNEL-positive (apoptotic cells). After calculating the apoptotic neurons against the observed neurons, there was no significant difference between the apoptotic rate of FPF treatment groups at 4 mg/L and the control group (*p* > 0.05), while the apoptotic rates of other treatment groups (8 mg/L, 20 mg/L, 50 mg/L, and 125 mg/L) were 26–30 folds and were significantly higher than that of the control group. There were no significant differences among the last four treatment groups (Figure 1C).

### Different expressions of genes related to immune pathways and detoxification in nurse bees after FPF exposure

**FIGURE 2:**
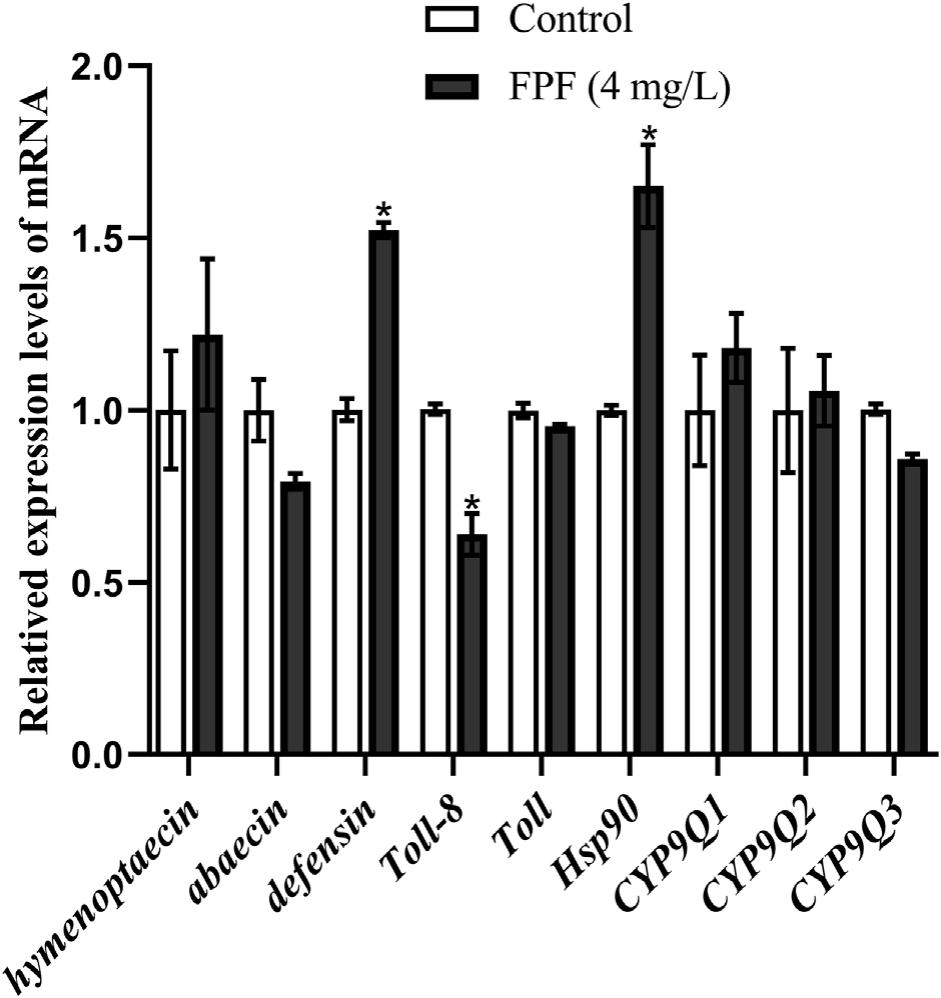
Relative expression levels of mRNA.

There was no significant difference between the 4-mg/L treatment group and the control group in the apoptotic assay, while this concentration was that to which bees could actually be exposed to. The relative expression of nine genes related to immune pathways and detoxification at this FPF concentration in nurse bees was measured by qRT-PCR. *Hsp90* and defensin were significantly upregulated, which were 1.65 folds and 1.51 folds higher than those of the control groups, respectively. *Toll-8/Tollo* was significantly downregulated, which was 0.63 times less than that of the control group. There were no significant differences between the expression of the rest genes, including *Tollo*, two antibacterial peptide genes (*hymenoptaecin and abaecin*), and three detoxification genes (*CYP9Q1*, *CYP9Q2*, and *CYP9Q3*), and the control groups after FPF exposure at the field-realistic concentration (Figure 2).

## Discussion

Due to the hypothesis that FPF is more toxic to nurse bees than larvae, the FPF toxicity on two early developmental stages of bees (nurse bees and larvae) was compared through two acute oral tests. Mushroom bodies were the key target of nAChR for neonicotinoids (Bicker and Kreissl, 1994; Goldberg et al., 1999; Déglise et al., 2002). FPF has a similar mechanism of action with neonicotinoids in the mushroom bodies of insects (Nauen et al., 2014). Then, the TUNEL method was used to assess the effect of FPF on nervous apoptosis in mushroom bodies of nurse bees, using a field-realistic concentration of FPF that could be encountered in nature. Since FPF at this concentration had no significant effect on the mortality and apoptosis of nurse bees in the previous two assays, qRT-PCR was carried out to determine the different expressions of immune and detoxification genes in nurse bees induced by FPF at the field-realistic concentration.

### Nurse bees were more sensitive to FPF than larvae

A total of five different FPF concentrations were exposed on *in vitro* rearing of bee larvae (4 days after hatching), and the results here were that the acute oral LD_50_ was 17.72 μg a.i./larva. Another five different FPF concentrations were fed to nurse bees (7 days after emergence) in the laboratory, and the value of acute oral LC_50_ was 125.92 mg/L; the corresponding LD_50_ was 3.368 μg/bee (3,368 ng/bee) in this study. Therefore, the nurse bees were more sensitive to FPF than larvae, which was consistent with other studies where larvae were more tolerant to imidacloprid, thiamethoxam, and formetanate than adult honey bees (Yang et al., 2012; Laurino et al., 2013; Tavares et al., 2015; Dai et al., 2017; Staroň et al., 2017). Larvae had more insect fat body than nurse bees, which played an important role in an innate immune response, and had less Kenyon cells than adults, which were the main targets of neurotoxic pesticides (Farris et al., 1999; Blacquière et al., 2012; Palmer et al., 2013; Staroň et al., 2017; McAfee et al., 2022). These might account for the different sensitivities between nurse bees and larvae. Moreover, imidacloprid-exposed nurse bees showed less activity and social interaction (Medrzycki et al., 2003; Forfert and Moritz, 2017). Nurse bees were specifically critical for honey bee larvae, which relied on nurse bees for their growth and survival (Lindauer and Watkin, 1953; Metz et al., 2021). Sufficient larvae and nurse bees were essential to maintain colony strength and size. Thus, significant attention should be paid to nurse bees to maintain the fitness of the bee colony.

### The toxicity of FPF on bees was lower than some of the widely used pesticides

In our previous study, the LD_50_ value was 4.17 μg/larva for imidacloprid, 5.65 μg/larva for acetamiprid, 4.17 μg/larva for carbaryl, 14.83 μg/larva for amitraz, 0.46 μg/larva for chlorpyrifos, 2.70 μg/larva for coumaphos, and 0.83 μg/larva for fluvalinate(Dai et al., 2017; Yang et al., 2019), and Kim et al. (2022) found that the values of sulfoxaflor and cyantraniliprole were 11.404 and 0.047 μg/larva, respectively. LD_50_ for FPF on larvae here was observed to be higher than that of the common pesticides listed previously. Moreover, the NOED (no observed effect dose) of repeated FPF exposure on larvae was 1.32 μg a.i./larva (EFSA, 2015). Therefore, the larvae (*A. mellifera*) showed more tolerance to FPF than some of the widely used pesticides.

The LD_50_ value for FPF observed in this study was consistent with a value of 2.995 μg/bee reported by Tosi and Nieh (2019), which was 2.5–2.8 times higher than the value (1.2 μg/bee) reported by the US EPA (Glaberman and White, 2014). Previous studies have reported oral LD_50_ for imidacloprid that ranged from 3.7 to 40.9 ng/bee (Schmuck et al., 2001), with a highly toxic value of 5 ng/bee reported by Suchail et al. (2010). Also, the corresponding value for clothianidin was 3.53 ng/bee and that for thiamethoxam was 4.40 ng/bee in the study of Laurino et al. (2013). Similar to the results of the larval assay in this study, FPF had less acute oral toxicity to adults when compared to some other common pesticides. Therefore, the application of FPF in the field with the same dose for pest control might cause less mortality of larval and adult bees than the other pesticides mentioned previously.

### FPF at a sublethal concentration significantly increased the apoptosis of neurons in nurse bee brains

In this study, the TUNEL method was used to detect the effect of FPF on the apoptosis of neurons (Kenyon cells) in the mushroom bodies of nurse bees. The results showed that there was no difference in the apoptotic rate between the FPF treatment group at a field-realistic concentration and the control group, in which the rate of nervous apoptosis in the two groups was less than 10%. However, the apoptotic rate of the FPF treatment groups at the other sublethal concentrations (8 mg/L, 20 mg/L, and 50 mg/L) and the medium lethal concentrations (125 mg/L) was significantly increased (26–30 times), when compared to the control group. Furthermore, the apoptotic rate increased significantly when the concentration began with 8 mg/L. It was observed that 8 mg/L might be close to the lowest concentration of FPF, which could induce massive apoptosis in honey bee mushroom bodies. In our previous study, significantly increased apoptosis in dose- and time-dependent manners was found in bees’ brains after exposure to imidacloprid, and it was related to the caspase-dependent apoptotic pathway and autophagy (Wu et al., 2015). The results suggested that neurotoxicity (e.g., FPF and neonicotinoid) at the sublethal dose/concentration might cause damage to the neurons within bees’ brains when it exceeded a certain threshold.

The mushroom bodies served as the cognitive center for olfactory information processing, storing, and retrieving, which was required for sensory integration, memory and motor control, visual navigation, olfactory association and context-dependent learning, and memory in adult insects (Farris, 2005; Peng and Chittka, 2017). Kenyon cells are the major neuronal component of the mushroom bodies. Pesticides that target cholinergic neurotransmission (including nicotinic receptor agonists neonicotinoids and FPF) can disrupt honey bee cognition and behavior related to the function of Kenyon cells in mushroom bodies (Goldberg et al., 1999; Déglise et al., 2002; Dupuis et al., 2011; Belzunces et al., 2012; Blacquière et al., 2012; Palmer et al., 2013). Given the important roles of nurse bees and their sensitivity to pesticides, the impact of FPF on the mushroom bodies in nurse bee brains needed further investigation.

### FPF at the field-realistic concentrations induced immune responses in nurse bees

The concentrations of FPF exposed to bees were mainly field-realistic (Glaberman and White, 2014; Tosi and Nieh, 2019; Harwood et al., 2022). Although FPF at the field-realistic concentration had no evident adverse effect in acute toxicity and apoptotic assay, *Hsp90* was significantly upregulated after 24 h of exposure, which was consistent with that in newly emerged bees after 12-h exposure of FPF (Wu et al., 2015) and 72-h exposure of nicotine (Rand et al., 2015). *Hsp90* (heat-shock protein 90) acted as a protective function under stress and positive conditions related to the immune response in insects. In addition, it had anti-apoptotic properties in tumor cells (Wojda, 2017; Xu et al., 2017). Thus, it suggested that honey bees might respond to FPF toxicity by upregulating *Hsp90* in this study. However, McKinstry et al. (2017) discovered that heat shock (as a stress) represses multiple immune genes (*defensin*, *hymenoptaecin*, and *abaecin*), and wounding the cuticle of the abdomen results in the decreased expression of multiple HSR genes (including *Hsp90*) in bees, while *defensin* was significantly upregulated in this study. The relationship between *Hsp90* and antimicrobial peptides, as well as apoptosis, in nurse bees should be determined in the future.

*Defensin* was significantly upregulated in our results, which was consistent with that of newly emerged bees exposed by FPF at the colony level. The expression of *defensin* and apidNT was significantly higher than that of the control bees (Guo et al., 2021). *Defensin* was reported to be regulated by the Toll pathway in bees (Lourenço et al., 2018). Akhouayri et al. (2011) reported that the production of AMPs in the *Drosophila* was negatively regulated by *Toll-8*/*Tollo*. *Toll-8*/*Tollo*, in this study, was significantly downregulated, when *defensin* was significantly upregulated. It suggested that nurse bees might use *Toll-8*/*Tollo* to negatively regulate the production of *defensin* (or other undetected AMPs), which was one of the immune responses to acute toxicity of FPF.

There were no significant changes in the expression of three P450-related genes (*CYP9Q1*, *CYP9Q2*, and *CYP9Q3*) in this study. On the contrary, the expression of *CYP9Q2* and *CYP9Q3* was significantly upregulated in bee foragers, which were fed by FPF for 6 days. In addition, the survival rate of larvae in FPF-treated colonies was significantly lower than that in control colonies (Guo et al., 2021). Tan et al. (2017) also reported that the exposure of FPF affected the learning and memory ability of adult bees (*Apis cerana*), which were fed with FPF at a field-realistic dose during the bee larval stage. Although some genes related to immune pathways and detoxification were not affected by acute toxicity of FPF here, attention should be paid to the effect on the nervous system, immune, and detoxification responses in bee larvae and nurse bees when exposed by repeated field-realistic or higher sublethal concentrations/doses of FPF.

## Conclusion

We found that nurse bees were more sensitive to FPF than larvae and that the acute oral toxicity of FPF on nurse bees or larvae was lower than some of the widely used pesticides. Although the apoptotic rate in the mushroom bodies was not affected by FPF at the field-realistic concentration (4 mg/L, 24 h), *Hsp90* and Toll pathway-related genes (*Toll-8*/*Tollo* and *defensin*) in nurse bees were significantly modified. Moreover, FPF at other sublethal concentrations (8 mg/L, 20 mg/L, and 50 mg/L) could induce significant apoptosis in nurse bee mushroom bodies. Therefore, the relationship between cytotoxicity, immune responses, and behavioral abnormalities of nurse bees induced by FPF at sublethal concentrations/doses needs reassessments.

## Data Availability

The original contributions presented in the study are included in the article/Supplementary Material; further inquiries can be directed to the corresponding author.
